# Elements of General Pathology

**Published:** 1842-10-01

**Authors:** 


Elements of General Pathology. By the late *John Fletcher,* M.D. Edited by *John J. Drysdale,* M.D. and *John R. Russell,* M.D.

This posthumous publication of the lamented Dr. Fletcher, may be considered as a continuation of his "Rudiments of Physiology," and will be read with great pleasure by all, who took an interest in the views he promulgated in that learned and valuable work. The volume now before us, although perhaps less striking in the novelty of its doctrines, and less profound in its researches, than its predecessor, is very valuable; and occasions deep regret that its highly talented author should have been so prematurely removed.

His philosophical mind,—his ardent and untiring zeal,—his extensive reading,—and the clear and sensible conclusions he deduces from his experiments and researches,—place him in the highest rank of the physiologists and pathologists of modern times.

The present work is compiled from Notes of Lectures left by Dr. Fletcher; and it is fortunate for his memory, as well as for the interests of medical science, that the task of arranging them has fallen into the hands of the Editors. They have not only succeeded in giving a clear and connected view of Dr. Fletcher's opinions, but have enriched the work by additional notes; which not only elucidate and confirm his doctrines, —but in many instances supply from the writings of continental and other pathologists, what was left defective or untouched by him.

The work is divided into three books, viz. on Etiology—Semeiology—and Therapeutics:—the first division occupying more than half the volume. In the first chapter, on the Remote Causes of Disease, there is nothing particular to arrest our attention. The author defines his views of the various predisposing and proximate causes of disease—of temperaments—habit of body and the effect of climate and of diet. He then proceeds to speak of the exciting causes, which operate in producing disease, only in conjunction with the predisposing causes, and through the medium of the proximate.

One of the most frequent exciting causes of disease is a variable temperature. Dr. Fletcher thus explains the manner in which heat and cold occasion disease.

Heat, by producing inordinate irritation exhausts the irritable matter, consequently the subsequent irritation even from the ordinary stimuli will be less than usual, and only slowly recover itself—hence inflammation, &c. On the contrary cold, which is nothing more than the absence of caloric, is a defect of the accustomed stimulus, and therefore gives rise to an accumulation of irritable matter, and irritability; which is followed by a greater than usual irritation from no more than the ordinary stimuli. Of the two conditions then necessary to produce a disease, cold may be said to operate in general by increasing the susceptibility, and heat always by increasing the stimulus: but the ultimate action of both is the same; disease resulting as much from the increase of one as of the other. This we may observe is a modification of Brown and Darwin's theory. One of the first changes induced in the system by inflammation, is an alteration in the character of the blood; this fluid exhibiting, when drawn, the buffy coat. This condition of the blood is the effect, and never the cause of inflammation. It results from an imperfect decomposition of the blood in the capillary arteries; so that the ingredients going to form fibrine, albumen and colouring matter, being imperfectly decomposed and passing into the radicles of the veins, communicates the characteristics of sizy blood. The opposite condition of the blood, as in nervous apoplexy, cases of sudden death, &c. where there is too little fibrine to effect its coagulation, may be accounted for, Dr. Fletcher thinks, by the converse of the preceding explanation.

> "If an overloading of the capillary arteries (in which the blood is naturally decomposed) tends to produce an abundance of these principles, an overloading of the radicles of the veins (in which the blood is naturally re-composed) might be supposed, *à priori*, by preventing absorption, calculated to produce a deficiency of them. Now such an overloading or congestion of the venous system always takes place in cases of sudden death, owing to the suddenly impeded action of the lungs, while that of the heart is undiminished, by which the blood is always accumulated in the veins. It appears therefore a simple and beautiful view of the matter to refer all superabundance of the principles to inflammation, all deficiency of them to congestion; and we may be satisfied that, except when the preternatural coagulation or alteration of the blood is produced directly by chemical agents thrown into it, this explanation is applicable." 41.

Dr. Fletcher combats, with success, we think, the doctrine which supposes the buffy coat in the blood to arise from some morbid matter with which it may be impregnated; and thus becoming the cause rather than the effect of the disease in which it displays itself. He is not so conclusive in his arguments against the blood undergoing other primary morbid changes, and hence conveying the materies morbi in some constitutional and other ailments: a doctrine which, at the present day, seems to be gaining ground. Dr. Fletcher's predilection for the doctrine of irritation and sympathy as the means by which disease is propagated, leads him to disbelieve the existence of peccant matter in the blood, whether it be induced by primary changes in that fluid itself, or be the result of absorption.

We are not the advocates of any exclusive opinions, yet we so far incline to the doctrines of the humoral pathologists, as to believe that they frequently lead to successful treatment. In gout and gravel for instance (diseases which Dr. Fletcher brings forward in support of his own views) we apprehend the blood, either from repletion, impaired digestion or other causes, acquires a vitiated character, and the most successful treatment consists in changing, or eliminating from the system, these peccant causes.

The third Chapter, on Light, Electricity, and Air as Causes of Disease; contains some valuable observations on the nature of contagion and infection.

The author properly restricts the former term to those morbid emanations which are generated by the bodies of the sick; while the latter is applied to those miasms which arise from marshy grounds and other causes. It must be confessed that the knowledge we possess of the nature of the miasms of contagion and infection, is very limited, our information is principally confined to an investigation of their laws and effects. Dr. Fletcher is of opinion that dead and putrid animal or vegetable matter does not generate infectious miasms. The malaria arising from marshes and close damp woods, he attributes rather to diseased living vegetables than putrefying animal or vegetable substances. "The state in which vegetables exist in marshes and close wet woods is manifestly unhealthy and similar probably to that in which the animal body exists when labouring under a contagious disease; and as the animal body when dead ceases to put forth these miasms as its other secretions become less virulent, a fact well known to dissectors, so the vegetable body when dead ceases to emit any noxious exhalations." 69.

With respect to the manner in which contagious or infectious matters enter the body in order to produce their effects, there are three principal opinions, viz. that they do so either by the lungs, the stomach, or the skin. The first is the most ancient conjecture and is advocated accordingly by Lucretius: it has been supported also in recent times by Fermelius, Sanctorius, and lately by Sir A. Cooper.

The second was likewise an ancient notion and has been advocated recently by Lind, Darwin and Jackson. The third opinion seems to have originated with Fracastor, and has had but few advocates since his time. Our author inclines to the first of these opinions, and in the main we coincide with him. Our knowledge of the laws of contagion is however still too imperfect to enable us to dogmatise, or form any exclusive opinions on the subject.

Dr. Fletcher's advocacy of the doctrine of irritation and sympathy leads him to the conclusion, that it is not necessary that the contagious miasms be absorbed into the system to produce their effect.

"We have," he says, "no evidence whatever that such miasms ever require to be absorbed into the blood to produce their effect. Nay it is not improbable that such an absorption, if it really took place, would rather diminish than increase their action. This subject will be adverted to afterwards, but in the mean time we may observe that the most rational opinion of their mode of action (like that of heat, cold, &c.) is, that they produce in the parts exposed to them merely an irritation of a certain character, which being conveyed by sympathy to the organ on which chiefly they are to display their effects, excite there a secondary irritation, in which the disease consists." 80.

The Editors subjoin, in a note to this Chapter, some curious researches of Professor Heule of Zurich, on the essential nature of miasms themselves.

The view taken of this subject by Heule is that the miasmatic contagious diseases (corresponding to the purely contagious, and to those both contagious and infectious of Fletcher) depend for their existence and propagation on certain parasitical organized beings, or their germs, whose presence and development in the body are the exciting cause of the symptoms which constitute these diseases.

This subject is very interesting, but we must refer to the work itself, as well as to the researches of Professors Heule and Liebig, for its further elucidation.

In the next Chapter, on Aliment, there are many sensible remarks on the tendency of certain articles of diet and habits of life to produce disease.

Laryngitis is said to be a consequence of frequent dram drinking: cynanche pharyngea, inflammation and scirrhus of the gullet, often result from liquids taken too hot: while acid or cold liquids taken while the body is hot, are a common cause of gastro-enteritis. "With respect to gout it is unquestionably a frequent consequence of intemperance in the use of animal food and fermented liquors, and (but not in the way commonly supposed) not owing to any vitiation of the blood." 95.

In a note appended to this extract our author states his opposition to chemical and humoral doctrines, and, as it contains a summary of his mode of reasoning and views on this subject, we will quote it. We do not, however, quite coincide with him: we think the hypotheses he impugns are not so wild as he imagines, and that he occasionally "leaps to conclusions," without a careful induction of facts.

"This doctrine (the vitiation of the blood in gout, &c.) originated with the chemical pathologists, who chose to represent the blood in this disease as adulterated with a tartaric, at other times with a bilious salt; sometimes with an alkali, at others with an acid. Cullen has the merit of having done much to abolish this absurdity, but since the unfortunate discovery in 1797 that the calculi deposited in the bursæ mucosæ in this disease, contained lithic acid, which acid unluckily contains nitrogen, it has again been confidently presumed that gout (like nephritis and urinary calculi) arises from an abundance of this principle; and that animal food is instrumental in producing it, by affording this principle in excess; and this view of the matter seems to be backed by the old observation, that gout and gravel are frequently combined. It is needless to repeat here the numerous objections already stated to this wild hypothesis, as well as to that founded upon it, and supported by many 'leapers to conclusions' among ourselves, that it consists in an abundance of ready-made lithic acid in the blood. In Asiatic cholera the blood has by some (Dr. Stevens, &c.) been represented as primarily affected,—turned into an infected jelly for one thing, and of a prodigiously dark colour for another; the latter change dependent, of course, on too little salt in the serum. These however, are not primary but secondary changes of the blood, and arise from the depression of the powers of circulation, so that through the tardy flow of the blood through the parenchyma, its molecular changes are so sparingly effected that it undergoes a partial coagulation. The use of warm saline injections is not to dilute the jelly aforesaid, nor to impart salts, but merely to afford a transitory stimulus to the heart, and thus accelerate for a time the circulation." 96.

Dr. Fletcher's opposition to a vitiated condition of the blood being the cause of disease, leads him to doubt whether morbid poisons are generally absorbed: he thinks they occasion disease in the same manner as infectious miasms, viz. by exciting in the organ to which they are immediately applied, a peculiar irritation which, when conveyed by sympathy to the other parts of the body, produces its principal effects on that part which is most adapted by its specific irritability to be so acted upon.

In a very sensible note the editor clearly shews that Dr. Fletcher has greatly underrated the frequency of the absorption of poisons. They also subjoin a very interesting table shewing the various poisonous and other substances—such as quicksilver, arsenic, indigo, &c. &c. which have been detected in the blood, and others, such as copaiva, hyoscyamus, &c. which have been found in the excretions, and must of course at one time have been contained in the blood.

In the chapter on Sympathy and the Passions as influencing Diseases, Dr. Fletcher adopts the idea we contend for—that exclusive principles of reasoning will not apply to medicine. For instance, it has been concluded that, because fever very frequently arises from local inflammation it necessarily always does so—and since the appearance of Broussais' works every question with respect to diseases in general has been answered by some certain pathologists with the single word "gastro-enterite." In exposing however the sweeping conclusions of others, Dr. Fletcher does not seem to be aware that he is sometimes liable himself to the same imputation: his conclusions are occasionally warped by his preconceived theory, and his practical deductions made subservient to it.

One of our author's hobbies we need scarcely remark, is sympathy. "It is by means of sympathy alone that all the exciting causes of disease already mentioned, *when they produce diseases in parts at a distance from those to which they are directly applied,* must be presumed to operate, and it is on this principle that many of the effects of heat and cold, of the numerous corruptions of the air, of various kinds of aliments, and of several poisons, have been already explained, and we shall have occasion to speak of the same agent in explaining that of some other exciting causes of disease, as well as of many remedies by which diseases are removed." 117. Dr. Fletcher then goes on to notice the influence of sympathy, as it regards the production of one disease from another. Among the two or three familiar instances he adduces as proofs that diseases are propagated by sympathy, is inflammation of the eyes in gonorrhœa. With respect to this intractable malady we believe that it is seldom, if ever, produced by sympathy. Our own observation would lead us to the conclusion that it is the result of the actual contact of gonorrhœal matter, applied to the eyes by the fingers involuntarily, and even during sleep. In this opinion we believe we are borne out by Lawrence, Jacob, and other writers on the eyes: but we should not have noticed so apparently trivial a matter had it not been by way of caution: for the disease may almost invariably be prevented by timely care on the part of the patients.

In the 6th and 7th Chapters, on Accidental Stimuli, the Excretions, Exercise, &c. as Causes of Disease, there are many sensible practical remarks, but we do not observe any striking novelty. We agree with Dr. Fletcher that copious bloodletting and hæmorrhage are frequent causes of disease, and great praise is due to Dr. Marshall Hall for the care with which he has investigated this subject.

Were we disposed to cavil at Dr. Hall, we should say that many of his distinctions are somewhat too refined, and the contra-indications about bloodletting not sufficiently clearly pointed out. In the treatment of puerperal diseases, for instance, we question whether they may not occasionally deter practitioners from the use of the lancet, when the safety of the patient depends on it. We entirely concur, however, in the sound practical information embodied in the following quotation from the work before us.

"The instrumentality of copious hæmorrhage or blood-letting in producing puerperal fever was insisted on by White and Mannering, and its effects in occasioning dropsy have been observed from the earliest time. Good, also Abercrombie, M. Hall and many others, have distinctly traced hydrocephalus to that cause; and it was proved many years ago by Kellie and Saunders, that by bleeding animals to death, they always produced a deposition of serum in their brain.

That the insanity of puerperal women also often arises from the hæmorrhage which they have undergone, is proved by Good, M. Hall, and many others; and that a kind of delirium tremens with constant watchfulness has often (particularly in young persons) this origin, is well established, so also that epileptic convulsions often arise from this cause is abundantly well known, and perhaps this is not unfrequently the origin of those which so frequently occur during parturition.* In addition to these, amaurosis has been observed to arise from this cause by Richter and Travers; and nervous apoplexy is as well known a result of it in the old, as delirium tremens in the young. The explanation of all this is sufficiently easy. With respect to the organic diseases above mentioned, their essence—not less the dropsies than the fevers,—is inflammation: and how readily inflammation is excited by a want, as well as by an excess, of any ordinary stimulus is well known. If cold then can produce this state, we should *a priori* say that hæmorrhage may do so too.

Nor with respect to the functional diseases is the difficulty greater. In the case of insanity it acts obviously on the vessels of the brain, so as to produce irregular circulation; and it is still through the brain, in all probability, in the case of syncope in which the heart principally suffers, in delirium tremens and nervous apoplexy, which involves both the mental faculties and the voluntary motions, and in that of epilepsy which involves the latter only. 'En privant,' says Andral, and most truly, 'le cerveau de ses excitants accoutumés on peut produire précisément ces mêmes effets que ceux auxquelles on donnerait lieu en augmentant la quantité de ses excitants.'"

The Editors have appended to the 7th Chapter some very interesting results obtained by Professor Rokitansky, on the incompatibility of certain

* "One of the most common consequences of this exciting cause of disease is a kind of chronic syncope, similar to what is called mercurial erethism, displaying itself chiefly in a remarkable susceptibility to any impression and a tendency to syncope from the slightest causes." (Pearson, Cooke, &c.)

diseases with each other. From the earliest ages a vague idea has prevailed that two diseases could not co-exist in the system. This opinion was thus far modified by John Hunter, who says that "no two actions from two different morbid poisons can go on together at the same time, in the same part, or the same constitution." Later observations, while they have shewn this statement, as expressing a general law to be erroneous, have at the same time indicated, that certain diseases exert upon others an opposing influence, in the way of the one arresting the course or modifying the nature of the other. For example, measles and small-pox have been observed to suspend, or modify the course of each other. Hooping-cough sometimes suspends the small-pox, measles and scarlet fever. Hooping-cough is frequently cured by vaccination. It is sometimes also cured by small-pox and measles. Vaccinia may suspend, or in its turn be suspended by scarlatina. The plague was arrested by the prevalence of small-pox, but broke out again on its disappearance, according to Baron Larrey.

But these and other isolated facts are not of a sufficiently definite character to have attracted much attention; and it remained for Professor Rokitansky, whose unequalled opportunities of observation and acknowledged accuracy create the most perfect confidence in his investigations, to put this matter wholly in a new light, by establishing from an amount of cases that renders fallacy in the result, almost impossible, that certain diseases never co-exist; as the presence of the one arrests the progress, or prevents the occurrence of the other. The typhus abdominalis, *i. e.* with formation of the characteristic typhous matter, and which by Rokitansky is always understood under the name of typhus, is excluded by the various forms of puerperal fever. In 200 dissections of puerperal fever he did not find one complication of the typhous process. This immunity from typhus is given by the pregnant state, child-bed, and even, though in a less degree, by suckling.

Typhus and cholera, and typhus and dysentery are said to have the power of mutual exclusion: and the co-existence of tuberculous disease and typhus is extremely rare. Carcinoma and tuberculosis (*i. e.* tuberculous disease) are antagonist diseases: and the latter and all kinds of serous cysts are never met with simultaneously in the same organ, or even in the same individual. Tubercular disease affords an immunity from cholera, dysentery, hypertrophy of the heart, curvature of the spine, dilated bronchia, and almost all chronic diseases of the stomach. Tuberculosis and aneurism do not co-exist, and Rokitansky as well as others has remarked that the development of tubercle is arrested, although the disease is not subdued, by the pregnant state, as likewise by all large tumours of the abdomen. These conclusions are derived by Rokitansky from numerous post-mortem and other examinations, and although exceptions may occasionally occur, yet if their main truth be established by subsequent researches, they may be rendered available in the history and treatment of various affections: and furnish a text for more extended enquiries by future pathologists.

The three following Chapters on Inflammation, its Consequences, and Fever, are principally occupied with the history of the various theories which have been advanced on these debatable subjects. We have before noticed Dr. Fletcher's own views, as to the operation of those causes, such as heat, cold, &c. which occasion inflammation; the immediate effect of these causes he thus states.

"This morbid change (inflammation) consists evidently in a preternatural dilatation by blood of the capillary arteries, veins and lymphiferous and chyliferous vessels constituting the parenchymatous tissue of a certain part, produced by either, first an increased and afterwards a diminished action of the vessels, or first a diminished, afterwards an increased, and again a diminished action, according as the exciting cause has been either positive and stimulant, or negative and sedative, the blood becoming accumulated in them, not because they contribute less to its propulsion (for their action in this way is perhaps unnecessary), but because they are less able to resist the impulse with which it reaches them, and their calibre therefore becomes increased. They probably transmit neither more nor less blood than in a state of health; but owing to the increase of calibre, it of course traverses the vessels with less than its accustomed velocity." 157.

This simple view of the matter comes probably as near the truth as any of the multifarious theories that have been propounded on this subject; and the Editors subjoin a very long and elaborate note confirmatory of it, from the writings of various continental pathologists.

The doctrines with respect to the nature of fever have at all times depended more or less on those which were prevalent respecting inflammation; and Dr. Fletcher's theory does not falsify this assertion. He thus states it:

"This morbid change (fever) consists equally evidently in a preternatural dilatation of the capillary vessels of the whole surface of the body, produced always (at least when it arises from local inflammation) by, first an increased and afterwards a diminished action of these vessels, since the immediately exciting cause of fever in this case (viz. sympathy) operates always as a stimulus." 174.

The five subsequent Chapters are devoted to the consideration of the Products of Inflammation; and contain many ingenious speculations on the origin of tubercle, cancer, calculi, and other diseased formations.

Dr. Fletcher regards inflammation as the cause of most of the morbid products and diseased tissues of the body. In tracing tubercle, scirrhus, tumors, &c. and the various morbid fluid secretions to this cause, we believe he is supported by well-grounded argument; but we must pause before we admit his conclusion, that calculi, worms and other parasitical formations are usually, if not invariably, the result of inflammation. We have not space however to detail, or enter fully into, Dr. Fletcher's arguments on these controverted subjects. His opinions however are stated with great clearness and perspicuity, and he displays considerable critical acumen in detecting and combating the errors of former writers. A short Chapter on Spasms and Spasmodic Affections, concludes this, the etiological portion of the work.

*(To be concluded in our next.)*

